# Understanding humoral immunity and multiple sclerosis severity in Black, and Latinx patients

**DOI:** 10.3389/fimmu.2023.1172993

**Published:** 2023-05-05

**Authors:** Kiel M. Telesford, Lilyana Amezcua, Lauren Tardo, Lindsay Horton, Brett T. Lund, Anthony T. Reder, Timothy Vartanian, Nancy L. Monson

**Affiliations:** ^1^ Department of Neurology, Brain and Mind Research Institute, Weill Cornell Medicine, New York, NY, United States; ^2^ Multiple Sclerosis Comprehensive Care Center, University of Southern California, Los Angeles, CA, United States; ^3^ Department of Neurology, Keck School of Medicine, University of Southern California, Los Angeles, CA, United States; ^4^ Department of Neurology, University of Texas Southwestern Medical Center (UT), Dallas, TX, United States; ^5^ Department of Neurology, University of Chicago, Chicago, IL, United States

**Keywords:** multiple sclerosis, humoral response, ethnicity, B cell, autoimmunity

## Abstract

People identified with Black/African American or Hispanic/Latinx ethnicity are more likely to exhibit a more severe multiple sclerosis disease course relative to those who identify as White. While social determinants of health account for some of this discordant severity, investigation into contributing immunobiology remains sparse. The limited immunologic data stands in stark contrast to the volume of clinical studies describing ethnicity-associated discordant presentation, and to advancement made in our understanding of MS immunopathogenesis over the past several decades. In this perspective, we posit that humoral immune responses offer a promising avenue to better understand underpinnings of discordant MS severity among Black/African American, and Hispanic/Latinx-identifying patients.

## 1 Introduction

Twenty years of observational studies demonstrate that relative to those identified with White ethnicity, people identified with Black/African American, or Hispanic/Latinx ethnicity are more likely to experience rapidly disabling multiple sclerosis (MS). MS is a chronic inflammatory demyelinating and neurodegenerative disease of the central nervous system (CNS). The pathologic hallmarks of MS include accumulation of focal inflammatory demyelinating lesions in the white matter, atrophy of cortical and deep gray matter, and the presence of oligoclonal bands in the cerebrospinal fluid (1). Diagnosis is based on clinical and radiological findings that support dissemination of lesions in space and time. Approximately 80-85% of MS patients are initially diagnosed with relapsing remitting MS, characterized by discrete episodes of neurological dysfunction followed by partial, complete, or no remission (2). Over time, gradual worsening occurs in the absence of relapse, termed secondary progressive MS.

The few studies examining potential factors that mediate discordant MS severity among Black/African American, and Hispanic/Latinx identified people focus primarily on social determinants of health, such as measures of socioeconomic status. However, the potential contribution of established immunobiological mediators of MS pathophysiology to this discordant severity have scarcely been reported. An emergent immunopathic role for B cells in MS, and immunologic studies across several fields, strengthens the importance of understanding humoral immunobiology for novel translational insights. While the available published data exploring the relationships between immunobiology and this discordant MS severity is sparse, antibody production has been highlighted as a potentially important factor.

In this review, we distill 1. the incidence of MS among Black and Hispanic/Latinx populations, 2. The key clinical features of MS severity among Black, and Hispanic/Latinx-identifying people; 3. The key radiological features of MS severity among Black and Hispanic/Latinx-identifying people, 4. The evidence of pronounced humoral immune responses in Black, and Hispanic/Latinx-identifying people in the contexts of infection, autoimmunity, and MS; and 5. The present limitations as well as opportunities for future study of discordant MS severity among Black, and Hispanic/Latinx people.

For this review, we employ the terms ‘Black/African American’, as well as ‘Hispanic/Latinx,’ acknowledging that they are socially constructed identity categories that allude to a spectrum of cultural, geographic designations, and nationalities (3). We also note that there are several studies highlighting MS incidence and clinical presentation of patients identifying with additional cultural, and national identities such as indigenous populations, individuals from the Middle East, Japan, and others (4–6). This review focuses on the sizable and growing body of literature that specifically compares ‘Black/African American’, ‘Hispanic/Latinx,’ and ‘White’ MS patient trends, primarily in the US context.

Our review of the literature encompassed clinical, as well as translational immunologic studies comparing outcomes between participants identifying with ‘Black/African American’, ‘Hispanic/Latinx,’ and ‘White’ ethnicity categories. These studies included indications such as MS, systemic lupus erythematosus (SLE) as well as health status contexts of infection and public health. Our literature search was performed on PubMed using search terms that included the above ethnicity categories, along with the listed indications, as well as: ‘incidence’; ‘prevalence’; ‘severity’; ‘ancestry’; ‘MRI’; ‘atrophy’; ‘IgG index’; ‘immune response’; ‘inflammation’; ‘lymphocyte’; ‘B cells’; ‘T cells’; ‘risk allele’; as appropriate. For studies involving other related health statuses or public health related investigations, we employed search terms that included; ‘serology’; ‘seroprevalence’; ‘vaccine response’; ‘socioeconomic status’; ‘obesity’; ‘social determinants of health’; ‘disparities research framework’.

## 2 Incidence of MS among Black and Hispanic/Latinx populations

Despite the widely held belief that MS is a condition that primarily affects White-identified people with European descent (7), recent MS incidence and prevalence rates demonstrate the importance of taking ethnicity into account during clinical investigation. A study of MS incidence among 2,691 Gulf war veterans revealed that 651 individuals reporting Black/African American ethnicity had an increased risk of developing MS [12.1 cases per 100,000, (CI 11.2-13.1)] relative to 1,792 individuals of White ethnicity [9.3 cases per 100,000, (CI 8.9-9.8)]. A separate incidence study of Southern Californian patients corroborated these trends: 106 Black/African American patients,10.2 cases per 100,000; 258 White patients, 6.9 cases per 100,000; and 116 Hispanic/Latinx patients, 2.9 cases per 100,000 (8–10). Overall MS prevalence among this same patient population revealed comparable proportions between Black/African American [225.8 cases per 100,000 (CI 207.1-244.5)], and White-identified patients [237.7 cases per 100,000 (CI 228.2-247.2)], with a substantially lower prevalence among Hispanic/Latinx patients [69.9 cases per 100,000 (CI 64.4-75.5)] (11). This data, alongside the overall increasing prevalence of MS in the US, emphasizes the importance of ethnic-diversity in research cohorts to understand various confounders contributing to MS burden.

## 3 Clinical measures of severity of MS among Black, and Hispanic/Latinx patients

Clinical studies over the past twenty years demonstrate that both Black/African American, and Hispanic/Latinx MS patients exhibit a greater risk of severe disability, faster disease progression, and faster rate of evolution to secondary progressive MS, among other features (**Table 1**) (12, 13, 15, 19–21). Other notable observed trends include both clinical presentation, as well as radiological measures of lesions and atrophy. The majority of these investigations focused on study participants identifying with Black/African American ethnicity. Studies examining differences in clinical or radiologic severity involved cohorts comprising participants on various disease modifying therapies.

**Table 1:** Clinical or Radiological Feature relative to White-identifying study participants.

	Black/African American	Hispanic/ Latinx	On DMT
**Higher proportion of females** (9, 12, 13)	✓	✓	NR
**Younger age at onset** (14)		✓	NR
**Earlier age at diagnosis** (9, 12, 13)	✓	✓	NR
**More likely to have transverse myelitis** (15, 16)	✓		NR
**More likely to have optic neuritis** (17, 18)		✓	NR
**Greater disease severity** (12, 19)	✓	✓	Yes^1^
**Faster transition to secondary progressive MS** (20)	✓		Yes^1^
**Higher MS incidence** (8, 9)	✓		NR
**More rapid disability accumulation** (12, 15, 21)	✓	✓	Yes^1^
**Greater lesion volume** (22–25)	✓	✓	Yes^1^
**Increased lesion number** (23)	✓		Yes^1^
**Greater retinal nerve degeneration** (25, 26)	✓		Yes^1^
**Greater brain atrophy** (24, 25, 27–29)	✓	✓	Yes^1^
**More pronounced spinal cord atrophy** (30)	✓		Yes^1^
**Greater mortality** (31)	✓		Yes^1^

‘✓’ indicates self-reported ethnicity category associated with the listed clinical or radiological feature; Blank cell indicates clinical or radiological feature is not associated with, or unreported for the self-reported ethnicity category indicated in the column.

‘DMT’ (Disease modifying therapy). NR = Not reported.

^1^ **Supplementary Table 1** lists DMTs reported in indicated studies

Some studies report differences in initial presentation, with Black/African American-identifying participants more likely to present with transverse myelitis, and Hispanic/Latinx-ethnicity being associated with an initial presentation of optic neuritis. Among those with clinically definite MS, expanded disability status scale scores (EDSS) at diagnosis were ~40% greater among 79 Black/African American patients compared to 80 White patients (EDSS 2.9 vs 1.8, p<0.0002), in a St. Louis, Missouri study cohort (21). At four-to-six years at follow up, EDSS scores for the Black/African American cohort were approximately twice that of White participants (EDSS 5.7 vs 2.9, p<0.0008). Severity score metrics in several other cohorts echo these trends, demonstrating a measure of generalizability to the disparate clinical observations. A multicenter MS cohort of 391 Black/African American study participants exhibited elevated severity scores compared to 1,255 White participants (pMSSS=4.5 vs 3.4; p<0.0001) (12). This finding parallels observations in larger registry studies with patients from New York (13, 19) and across multiple regions in the US (15).

High severity scale scores (i.e. EDSS = 6 or 7) correspond to substantial ambulatory disability and requirement of ambulatory assistive devices. Accordingly, time-to-cane occurred 6 years sooner (p<0.0001) and time-to-wheelchair dependency 8 years (p=0.05) sooner among 375 Black/African American patients compared to 427 White patients (15). Similarly, 30% of Black/African American study participants with MS (n=79), compared to 10% of White participants with MS (n=80), required wheelchair assistance at 12 years post diagnosis (21). These trends parallel data on transition to secondary progressive MS being more aggressive in Black/African American patients with MS compared to White patients. A cohort of Brazilian MS patients of African descent (n=33) transitioned to secondary progressive MS four years earlier than those identifying as White (n=117) (20). This result was reminiscent of a 3-year faster differential reported for 329 Black/African American MS patients from a New York MS patient registry (13).

Few studies examining ethnicity-associated differences in clinical disability or radiological measures during MS focused on or included Hispanic/Latinx-identifying participants. Nonetheless, these also uncovered trends for greater CNS atrophy, increased T2 lesion volume and increased disability relative to White-identifying participants. For instance, 258 Hispanic/Latinx exhibited greater severity scale scores (pMSSS=3.9) than 1,255 White-identifying participants (pMSSS=3.4). (p<0.0001)

## 4 Radiological characteristics of MS among Black, and Hispanic/Latinx patients

Similar to clinical disease course, radiographic heterogeneity exists across people with MS identifying across White, Black/African American, or Hispanic/Latinx ethnicity categories. Studies demonstrate that compared with White-identifying counterparts, Black/African American identifying people with MS exhibit greater volumes of both T1 and T2 weighted lesions, as well as lower magnetization transfer ratios (22, 23). Currently, it is unknown whether differences in the initial radiologic lesion manifestation exist across these ethnicity groups. More recently, Black/African American-identifying people with MS have also been shown to exhibit higher atrophy rates of the medulla-upper cervical cord (30). The number of studies evaluating radiographic changes among Hispanic/Latinx-identifying patients with MS is small, but there is evidence that Hispanic/Latinx patients may experience greater rates of both global and regional brain volume loss when compared to White-identifying counterparts (24). While the overall number of studies investigating these radiographic findings across Black/African American, and Hispanic/Latinx ethnicity is sparse, they suggest that these patient populations do experience higher rates of neurodegeneration when evaluating volumetric MRI changes. Further longitudinal work is needed to better understand the potential radiological differences in minority patients with MS as well as the impact these changes have on disability.

## 5 Humoral immunity characteristics among Black, and Hispanic/Latinx patients

### 5.1 Primer on humoral immunity

**Figure 1:**
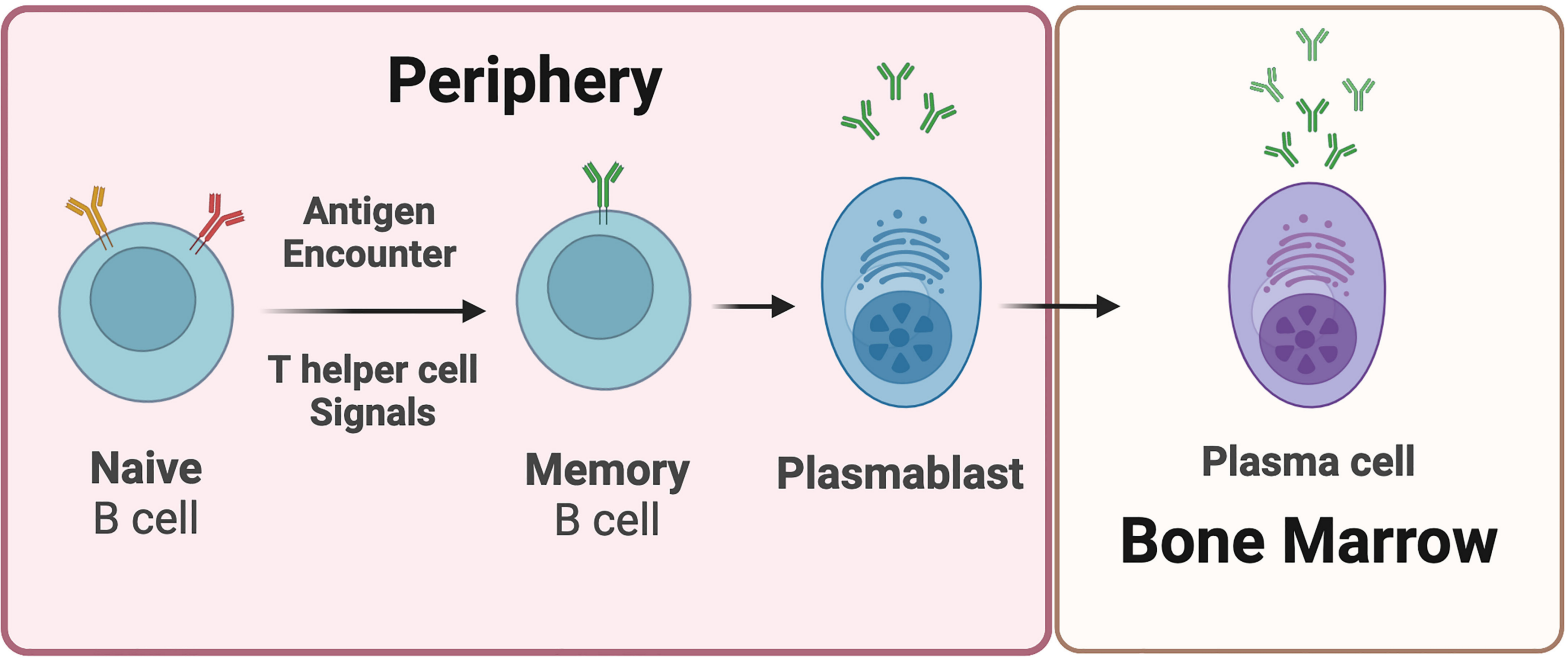
Plasmablasts and plasma cells are B cell-derived antibody-secreting cells. Upon antigen encounter, naive B cells receive signals from T helper cells and differentiate into memory B cells and plasmablasts. Plasmablasts are considered early-stage antibody secreting cells. Plasmablasts that migrate into the bone marrow are referred to as plasma cells. Plasma cells are relatively longer-lived, and undergo limited if any proliferation. Plasma cells are generally considered terminally differentiated antibody secreting cells.

Understanding the immunological underpinnings of MS is critical for advancing treatment strategies to stop progression and encourage repair. Innate and adaptive immune components are involved in the pathogenesis of MS, exhaustively reviewed elsewhere (32, 33). One hypothesis is that autoreactive helper T cells drive the autoimmune response associated with MS (34). These CD4+ helper T cells are activated by putative antigens expressed in the brain, and incite and sustain other cell types such as CD8+ T cells and B cells to participate in the autoimmune response (35). B cells in particular recognize their cognate antigen, become activated in a T-cell dependent manner and differentiate into antibody secreting cells (ASC’s) that produce antibodies against the activating antigen (36) (**Figure 1**). These principles have driven the field, highlighting that the majority of disease modifying therapies (DMTs) in MS focus on dampening or completely hindering an immune response in the CNS, although the first, and perhaps, primary action of most DMTs is in the periphery. Other immune response mechanisms in the CNS are reviewed elsewhere (37).

### 5.2 Ethnicity and humoral immunology; impact on infection and autoimmunity

Ethnicity is associated with differential susceptibility to infectious diseases (*i.e*., influenza, tuberculosis, dengue fever, HIV, measles, smallpox), adverse events following vaccination, and variations in immune response (38–42). For example in the setting of infection, Black/African American and Hispanic/Latinx-identifying individuals display significantly (p<0.0001) higher titers of anti-SARS-CoV2 antibody (~4.6 index Specimen/Calibrator values) compared to White-identifying individuals (~3.78 index Specimen/Calibrator values) in a study of 8,000 plasma donations (43).

In the setting of vaccination, people of Black/African American ethnicity display significantly higher (p<0.0001) rubella-specific neutralizing antibody titers (86.2) than those of White ethnicity (61.9) following vaccination (42). Age was also a determining factor driving ethnicity-associated differential B cell frequency and anti-influenza antibody trends after influenza vaccination (44, 45), highlighting the importance of accounting for multiple demographic variables.

In the setting of variations in immune response, ethnicity-associated differential immune responses are reported in serology for both auto antigens, and immunoglobulin levels among otherwise healthy individuals (46–48), and CD5 B cells in schizophrenia (49). Similarly, high rates of renal graft failure in Black/African American patients are attributed to high antibody-producing B cell frequency compared to White patients (50). The greater antibody levels often reported for people of Black/African American ethnicity could reflect ethnicity-associated differences in B cell signaling pathway dynamics (51). In influenza, a reduction of B- and T-lymphocyte attenuator (BTLA) expression in B cells is linked to higher antibody production in Black/African American individuals relative to White individuals (44).

Despite the paucity of reports that investigate relationships between ethnicity and molecular underpinnings of hyperresponsive B cells, studies in systemic lupus erythematosus (SLE) offer significant insight. SLE disproportionately affects people of Black/African American and Hispanic/Latinx ethnicity compared to people of White ethnicity (52) in incidence, and severity (53, 54). Also, like MS, B cells play a prominent role in promoting pathology. Careful longitudinal transcriptomic analysis of pediatric SLE patients demonstrates an activated, B cell-derived antibody-secreting plasma cell-like expression profile among Black/African American patients relative to patients of other self-reported ethnicities (55). This signature is a robust biomarker for disease activity, is more correlated with autoreactive antibody titers than other biomarkers, and parallels severity scale scores, which are greatest among Black patients in the analyzed dataset. Black/African American SLE patients may also have increased frequencies of highly activated CD86+ memory B cells (56) and reduced expression of FcgammaRIIB (57) (a noted component in the balance of tolerance and autoimmunity) (58) that renders B cells more prone to activation. While more severe SLE is also reported among Hispanic/Latinx-identifying people, cellular immune profiles remain under-reported (55). Of the 315 publications focusing on Hispanic/Latinx populations with SLE spanning nearly 3 decades, nearly all focus on prevalence, response to therapy, and some genetic risk assessments.

### 5.3 Ethnicity and humoral immunology; impact on MS

Delineation of ethnicity-associated differential immune responses in MS will likely refine our ability to identify and treat pronounced clinical, and radiological MS presentation among Black/African American, and Hispanic/Latinx-identified people. However, investigation into ethnicity-associated immunologic differences in MS is largely limited to risk allele studies (59–63). and do not explain heightened MS disability among Black/African American MS patients, compared to those reporting White ethnicity (63, 64).

Early evidence of B cell involvement in MS included 1) the prevalence of polyclonal antibodies in the cerebrospinal fluid of MS patients (27, 65–69), 2) activated memory B cells, plasmablast B cells and antibody secreting B cells in the cerebrospinal fluid and brain tissue that are likely from peripheral blood B cells (65, 66, 70, 71), 3) the ability of B cells to drive disease in a mouse model of MS (72), and 4) a heightened pro-inflammatory cytokine profile that drives pro-inflammatory T cell activation (73–75). These early studies prompted the use of B cell depletion treatment as a therapeutic approach to MS and has shown great efficacy (76–78). Based on the efficacy of B cell depletion therapy for MS, some have proposed that B cells play a central role in MS immunopathogenesis, with CNS-infiltrating peripheral blood memory B cells primarily driving acute lesion inflammation (76–78), while derivative ASCs contribute to gradual ongoing CNS degenerative processes. Many of these concepts have been demonstrated in mouse models of MS such as experimental autoimmune encephalomyelitis (EAE) which promotes an autoreactive lymphocyte response to myelin oligodendrocyte protein or other CNS antigens (79, 80).

Given the importance of B cells in MS disease activity, and the worse prognosis among Black/African American, and Hispanic/Latinx MS patients, identifying differential ethnicity-associated immune dynamics in MS may nuance considerations of MS immunopathogenesis. However, scant data outside of retrospective clinical or genetic (17, 61) analyses exist to understand the biology underlying ethnicity-associated disparity in MS severity. More than likely, a combination of genetics, environmental factors, behavior, and social determinants of health (81), -all capable of influencing immune responses- drive ethnicity-associated differential clinical severity. Yet, differential B cell activity appears to be an important biological correlate or contributor to disparate MS severity.

A handful of studies demonstrate that Black/African American MS patients have a greater CNS antibody burden (67, 82, 83) and robust intrathecal immunoglobulin responses compared to White MS patients (82). In a retrospective analysis, oligoclonal bands were present in the CSF of 87% of Black/African American MS patients (n=66) compared to 74% in White MS patients (n=132), while the median antibody (IgG) synthesis rate in Black/African American MS patients was 5.35 mg/day greater than White MS patients(13.55 mg/day vs. 8.2 mg/day p<0.01) (82). There was a positive association (χ^2 =^ 3.54; gl=1, p=0.051) between Brazilian MS patients of self-described African descent and the presence of OCBs (83). Finally, a chart review study compared IgG index in 300 Black and White MS patients in Detroit, Michigan (67). Despite similar disease duration and the interval between symptom onset and treatment, the 150 Black/African American MS patients exhibited significantly higher IgG index (1.50 vs 1.07 p<0.0005), relative to 150 White patient counterparts. Importantly, higher IgG index correlated with increased gadolinium enhancing lesions, grey matter atrophy, and spinal cord atrophy (67).

A second line of evidence stems from the general association between ASCs as key biological contributors to ethnicity-associated differential CNS pathology (71, 84–90) and clinical progression (84, 91–94). Intrathecal (CSF) ASCs are elevated in the presence of active lesions (71) and positively correlate with enhancing lesion volume in MS (r=0.441 p<0.001) (84). This suggests that the greater lesion number (23) and lesion volume (22, 23) exhibited by Black/African American MS patients relative to White patients may result from differential immunopathic ASC dynamics. Consistent with this notion, intrathecal antibodies strongly correlate with grey matter atrophy among Black MS patients (r= -0.893 p<0.00004) but not White patients (r= -0.019. p<0.85) after correction for age, T2 lesion burden, EDSS, and disease course (p<0.0005) (67).

**Figure 2:**
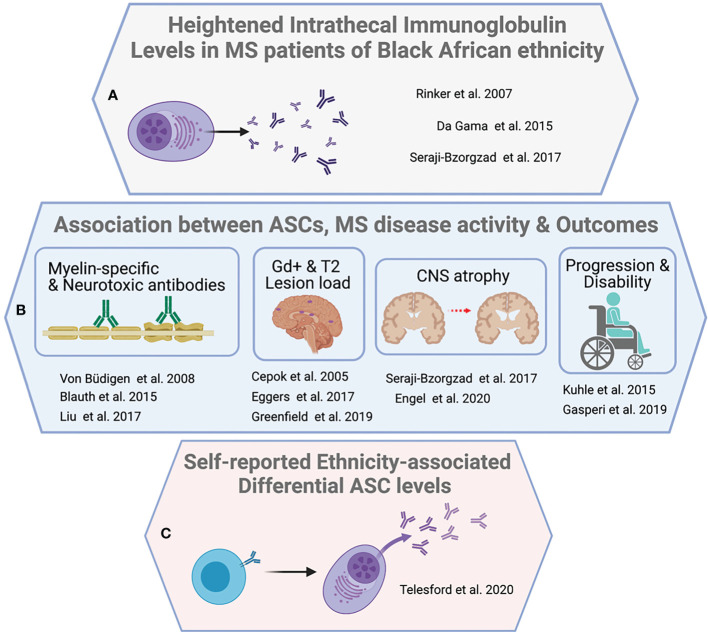
Evidence supporting B cell activity as a driver of ethnicity-associated MS severity. Three broader observations underpin an argument for the centrality of B cells in ethnicity associated severity disparity. **(A)** Reports of increased CSF lgG index, synthesis rate, or oligoclonal banding among individuals with MS identifying with Black/African American ethnicity. **(B)** Multiple associations between B cells, ASCs and antibodies with clinical, and paraclinical measures, as well as demonstrated CNS reactivity or neurotoxicity in vitro. **(C)** Inaugural direct demonstration of significantly increased circulating ASCs in MS patients of identifying with 'Black African' or 'Latin American' ethnicity relative to 'White' ethnicity.

A third line of evidence includes the only published investigation into differential ASC frequency in relapsing-remitting MS to date of self-reported Black/African American, and Hispanic/Latinx identity (95). The sample population included MS patients on natalizumab (‘Black’ n=17, ‘Latin American’ n=10, ‘White’ n=27), as well as those off of disease modifying therapies (‘Black’ n=8, ‘Latin American’ n=4, ‘White’ n=8); and a cohort of healthy participants (‘Black’ n=8, ‘Latin American’ n=3, ‘White’ n=13). Circulating ASC frequencies were significantly elevated among ‘Black, and Latin American’ participants with MS relative to White participants (natalizumab, 1.16% vs. 0.72% p=0.046; untreated, 6.56% vs 2.1% ASC p=0.042). While ethnicity–specific ASC frequency differences were observed in MS patients, they were absent in healthy donors (healthy participants, 1.6% vs. 2.11% ASC p=0.42) (**Figure 2**).

Beyond these lines of evidence, others recently note that Black/African American MS patients exhibit faster B cell reconstitution kinetics compared to White MS patients after B cell depletion (96). These data collectively suggest that there are MS-specific interactions between immune regulation and ethnicity. The observed greater antibody burden observed among Black/African American MS patients are likely the result of complex interactions between biological (allelic) as well as social (behavioral, environmental) relationships. Understanding these relationships may hold important translational insights to key underpinnings of disabling MS that occurs across ethnicity categories.

### 5.4 Opportunity for additional study of ethnicity-associated humoral dynamics in MS

The preceding sections demonstrate associations between Black/African American, or Hispanic/Latinx ethnicity and heightened humoral responses in several contexts. However, much of the immunobiology driving observed heightened humoral responses among these MS patient populations remains unstudied. For instance, there are currently no published data on differential T cell population dynamics, or function. As referenced in the preceding sections, T cells play prominent roles in MS incidence and pathophysiology. Current work provides indirect evidence of increased T helper cell activity in the form of heightened switched antibody responses (67, 82, 83, 95) among Black/African American-identified MS patients. Additional direct investigation of T cell dynamics, such as follicular helper cell subsets would add resolution to cellular interactions driving the observed increased humoral responses.

Presently published studies of antibody-mediated immunopathic effects such as demyelination have either not included or accounted for Black/African American, or Hispanic/Latinx -identifying patients in their analyses (87, 88, 97). Investigation of autoreactive antibodies, as well as potential defects in T regulatory cell-mediated peripheral tolerance (98) may lead to identification of new auto-antigen targets in MS, as well as insights into MS etiology and prognosis. Finally, most of the handful of published studies examining ethnicity-associated discordant humoral responses in MS, focus on participants identifying with the Black African diaspora. Very little published data exists on immunobiology among Hispanic/Latinx-identified MS patients.

Recent studies show greater MS severity is associated with alleles essential to T cell-dependent B cell responses. One such study using a multivariate model, describes a significant association between the degree of African ancestry at the broader HLA genetic locus, and patient-reported disability. Inclusion of established HLA-DRB1 risk alleles in their analysis did not modify the association, suggesting the mediating influence of one or more of the dozens of other non HLA-DR genes comprising the HLA locus (63). In addition, subsequent analysis demonstrated a significant association between the gene *tnfrsf1a* (situated outside of the HLA locus), and disability (64).

**Figure 3:**
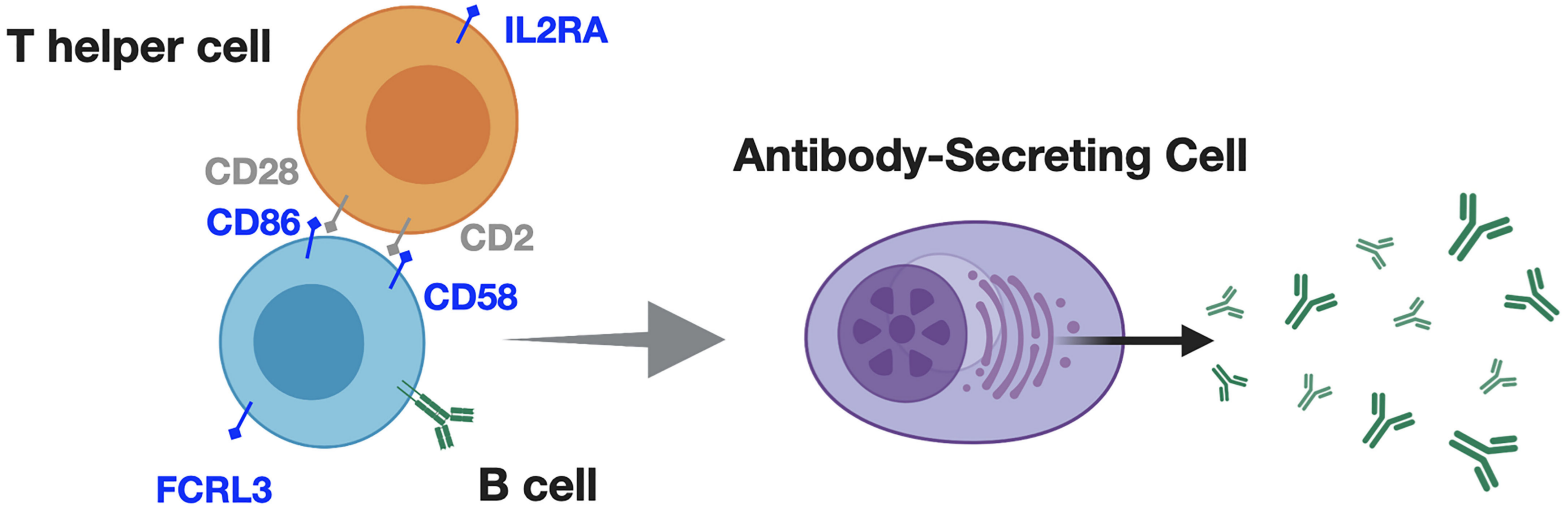
Alleles associated with T cell-dependent B cell responses may promote greater MS severity, in addition to greater MS risk for Black / African American identifying patients. T cells and B cells interact through contact and soluble mediators to produce downstream inflammation, and antibody responses. These responses appear to differ according to ethnicity in contexts of infection, and SLE. For example (blue text in figure), *IL2RA*; IL2 receptor alpha (CD25), which, is upregulated upon antigen-specific T cell activation, promoting IL2-mediated T cell survival and proliferation. CD2 on T cells interacts with B cell-expressed CD58, prompting inflammatory cytokine production, and antibody responses. CD86 provides 'signal 2' for T cell activation, while FCRL3 may regulate B cell receptor signaling, and differentiation into antibody-secreting cells. Particular alleles for these genes are associated with increased MS incident risk but their contribution to ethnicity-associated MS severity is undetermined.

Given the association between ethnicity, and varying levels of B cell activity both during infection, and in SLE, it is tempting to speculate that in the context of MS, alleles facilitating T cell-B cell interactions (99), or those regulating humoral responses (60) may promote heightened severity-for Black/African American or Hispanic/Latinx patients. Some genes, such as *IL2RA*, *CD58, CD86, and FCRL3* are involved in modulating T cell-mediated B cell responses and implicated in MS incident risk (59, 60). However, the contribution of these risk variants to ethnicity-associated MS severity is not established (**Figure 3**).

## 6 Present limitations, and considerations for future investigations

### 6.1 Underrepresentation of Black/African American, and Hispanic/Latinx-identified people in MS research

The dearth of available immunologic data across ethnicity categories in MS represents one of the most fundamental limitations to understanding discordant MS severity among Black/African American, and Hispanic/Latinx-identified people. This present lack of data is due in part to the gross underrepresentation of Black/African American and, Hispanic/Latinx-identifying people with MS in clinical and translational investigation. Despite Black/African American, and Hispanic/Latinx-identifying people representing 12%, and 16% of the U.S. population respectively (U.S. Department of Commerce. Overview of race and Hispanic origin: 2010 (U.S. Census Bureau—2010 Census Briefs), https://www.census.gov/prod/cen2010/briefs/c2010br-02.pdf) (100), publications specifically mentioning MS patients with these backgrounds reflects less than 1% of the literature (101).

Collective emphasis on the importance of including participants across ethnicity categories by funding agencies, and publishers may improve representation in clinical and translational studies. This may entail funding agencies prompting efforts to balance ethnicity during study recruitment. Peer reviewers, or journal editors may request clinical datasets include participants from different ethnicity background, highlighting the advantage of more representative datasets in comprehensively understanding MS, and advancing care. Additionally, greater awareness of MS registries may also improve participation by those underrepresented in current studies (102).

### 6.2 Underrepresentation of Black, and Hispanic/Latinx-identified people in MS clinical trials

FDA-approved DMT’s for MS have been assessed for safety and efficacy in cohorts consisting primarily of White participants. White ethnicity-identifying participants averaged 91.6% of those enrolled in 13 Phase 3 and Phase 4 clinical trials conducted between 2002 and 2020 (**Table 2**). By contrast, an average of 2.7% of Black/African American participants with MS were enrolled in these trials. Hispanic/Latinx ethnicity was not reported in 14 of the 17 trials listed here. MS patients from Black/African American, and Hispanic/Latinx backgrounds represent underserved populations with pronounced clinical need, considering the underrepresentation in Phase 3 and Phase 4 clinical trials (124) as well as the previously highlighted body of observational studies demonstrating greater MS disease severity. Moreover, Black/African American MS patients may be more likely to exhibit refractory disease while on interferon-beta (15, 125, 126), a common first-line MS therapy. Differing immune responses have also been noted among glatiramer acetate and natalizumab (127).

**Table 2:** Summary of phase 3 and phase 4 clinical trials including underrepresented populations.

Trial name	Therapy name	W | N (%)	B/AA | N (%)	H/L | N (%)
AFFIRM (103)	Natalizumab	603 (96%)	10 (1.6)	NR
SENTINEL (104)	Natalizumab + Interferon beta	1092 (93%)	39 (3.3)	NR
FREEDOMS I/II (105)	Fingolimod	NR	77(6.1)	** 181(4.96)
TRANSFORMS (106, 107),	Fingolimod	1216 (94.1)	NR	** 181(4.96)
TEMSO (108)	Teriflunomide	1058 (97.3%)	NR	NR
DEFINE (109)	Dimethyl Fumarate	969 (78.5%)	26 (2.1)	NR
CONFIRM (110)	Dimethyl Fumarate	1191 (84.1%)	27 (1.9)	NR
CARE MS I (111)	Alemtuzumab	532 (94.5%)	NR	NR
CARE MS II (112)	Alemtuzumab	714 (89.5%)	NR	NR
OPERAI/OPERA II (113)	Ocrelizumab	NR	72 (4.3)	NR
EVIDENCE (114, 115)	Interferon beta	308(91%)	36 (5.5)	NR
ESTEEM (116, 117)	Dimethyl Fumarate	616 (91%)	187 (3.8)	98 (1.96)
ASCLEPIOS I/II (118)	Ofatumumab	829 (88.6)	28 (2.9)	NR
*SUNBEAM (119)	Ozanimod	1340 (99.5)	2 (0.14)	NR
*CLARITY (120)	Cladribine	1300 (98)	7(0.5)	NR
*TOWER (121)	Teriflunomide	810 (89.8)	20 (2.2)	NR
*GALA (122)	Glatiramer Acetate	1371 (97.6)	15 (1)	NR
* ^#^ CHIMES (123)	Ocrelizumab	N/A	75 (50%)	75 (50%)

W, (White); B/AA, (Black/African American); H/L,(Hispanic/Latinx); NR, (Not reported); N/A (Not Available).

*Phase 4 clinical trials.

**H/L numbers are from pooled demographic data form three Phase 3 trials; FREEDOMS I, FREEDOMS II, and TRANSFORMS.

^#^Currently recruiting. Numbers represent recruitment targets.

Just as the use of targeted therapies improved understanding of MS immunopathogenesis, examining ethnicity-associated immunobiology may yield new insights into more effective treatments. Overall, variable efficacy of approved therapies using *post-hoc* analysis of ethnicity as a confounder requires particular attention, while highlighting the need for inclusive representation in trial design and *a priori* statistical analysis of efficacy. Indeed, the recent *post hoc* analysis (126) of Black/African American MS patients in Phase 3 trials of the B-cell depleting therapy ocrelizumab, suggested superior benefit for ocrelizumab compared to the interferon beta-1a arm across ethnic groups. Yet, Black/African American participants experienced suboptimal therapeutic effects on interferon beta-1a relative to White participants (twice the mean number of T1 lesions, and 72% greater T2 new/enlarging lesions per MRI scan during the study), consistent with earlier studies of interferon beta-1a that examined ethnicity-associated effects. However, more conclusive results were frustrated by the small number of Black/African American patients included in the overall study sample (**Table 2**), and by the limited pre-study data to account for preexisting differential clinical severity.

### 6.3 Recognizing and incorporating social and biological considerations in future study

Currently published work on ethnicity-associated discordant immune responses in MS emphasizes the biological domain of influence, at the individual level (128). More than likely, a combination of genetics, environmental factors, behavior, and social determinants of health (81), -all individually capable of influencing immune responses- drive ethnicity-associated differential MS severity. These factors exist across multiple domains of influence spanning interpersonal, communal, and even societal levels. Progress in understanding relationships between differential immunobiology, and ethnicity-associated discordant MS severity entails consideration, and analysis of data across social, biological and clinical measures.

In studies of MS severity for example, composite measures of socioeconomic status were demonstrated to modulate degeneration (129, 130) and disability (131) outcomes in Black/African American-identified patients. Socioeconomic status, and ethnicity also impact the access and utilization of specialty neurologic care (132). Discordant access to care, along with mistrust of biomedical research among Black/African American, and Hispanic/Latinx-identified MS patients (133) ultimately influences the participant composition within study cohorts, further influencing the samples collected for immunobiological study. Further, obesity (as measured by body mass index) is associated with lower socioeconomic status (134), and these factors can disproportionately impact Black/African American, and Hispanic/Latinx populations in the US. Measures of obesity appear to influence tissue degeneration (135–137), and disability (138) in MS, and these can also interact with humoral immunobiology (139).

Finally, additional care should be taken in how race and ethnicity data are collected, analyzed and reported in future work. The use of broader ‘race, and ethnicity’ groupings in several studies has revealed discordant severity as well as some immunobiological trends in MS. However, within the US, resident Black-, and Latin American-diasporas each comprise a range of nuanced sociocultural identities and ancestries (140). This heterogeneity is subsumed with the use of broad ‘race, and ethnicity’ groupings. Under a single grouping, such as ‘Hispanic or Latino’, nuanced identity combinations can vary between different US geographic regions. Presuming or homogenizing how individuals within Black/African American, or Hispanic/Latinx-diasporas identify in different contexts (141) increase the likelihood that more subtle clinically- and translationally- relevant insights may be missed.

## 7 Closing remarks

Heightened humoral immune responses may be responsible for increases in disease severity observed among Black/African American, and perhaps Hispanic/Latinx, MS patients. However, these observations lack depth, indicating that underrepresentation in both research studies and clinical trials evaluating effects of ethnicity remains an issue within MS research. Further, disparate severity, and treatment efficacy, may also be influenced by socioeconomic status-related influences associated with environmental and lifestyle factors. These may include behavior, diet, exercise, and smoking, in addition to genetic factors and ancestry.

To better address these discrepancies, we believe the most effective path forward requires: 1) outreach that equips Black/African American and Hispanic/Latinx patients with knowledge about MS, as well as the MS research that facilitates informed decisions surrounding participation in clinical and translational studies; 2) diversity within the MS clinical and research workforce which can enhance patient care and recruitment; 3) ensuring resources to aid greater generalizability in clinical trial sampling (142) that more accurately reflects the patient population; and, 4) research on ethnicity and genetic ancestry in conjunction with social and biological determinants of health.

Carefully controlled clinical and translational studies in MS that identify ethnicity, and ancestry-associated immunological differences, in combination with clinical trials in MS that achieve enrollment diversity, may illuminate cellular and molecular underpinnings of disparate clinical symptoms. These advancements may lead to breakthroughs in our understanding of MS pathogenesis and nuanced personalized treatments.

## Author contributions

KT, LA, LT, LH, BL, AR, TV, and NM each wrote or contributed to sections of the manuscript. All authors contributed to the article and approved the submitted version.
