# Supplementary material for: Understanding humoral immunity and multiple sclerosis severity in Black, and Latinx patients
Source: Front Immunol. 2023 May 5;14:1172993. doi: 10.3389/fimmu.2023.1172993 (PMC10196635; doi:10.3389/fimmu.2023.1172993)
Supplement: Supplementary file 1 [file Table_1.docx]

| **Supplementary Table 1: Disease modifying therapy (DMT) use in studies reporting ethnicity-associated discordant severity** | |
| --- | --- |
| **Clinical or Radiological Feature relative to White-identifying study participants** | **Specific DMTs among study participants** |
| Greater disease severity (12) | Specific DMTs not reported |
| Greater disease severity (13) | Specific DMTs not reported |
| More rapid disability accumulation (15) | Specific DMTs not reported |
| More rapid disability accumulation (16) | Specific DMTs not reported |
| Faster transition to secondary progressive MS (17) | Specific DMTs not reported |
| Greater lesion volume (18) | Interferon beta, glatiramer acetate, natalizumab, cyclophosphamide, mitoxantrone ^1^ |
| Increased lesion number (19) | Interferon beta, glatiramer acetate, natalizumab, azathioprine, mitoxantrone, intravenous immunoglobulin ^1^ |
| More pronounced spinal cord atrophy (20) | Dimethyl fumarate, fingolimod, ocrelizumab, interferon, natalizumab, dimethyl fumarate, glatiramer acetate, fingolimod, alemtuzumab, teriflunomide, ocrelizumab ^1^ |
| Greater brain atrophy (60) | Interferon beta, glatiramer acetate, natalizumab, rituximab ^1^ |
| Greater lesion volume (118) | Specific DMTs not reported |
| Greater retinal nerve degeneration (119) | Specific DMTs not reported |
| Greater brain atrophy (121) | Glatiramer acetate, fingolimod ^1^ |
| Greater Mortality. (122) | Specific DMTs not reported |
| 1 Duration of DMT varies among subjects | |
